# An effective protocol for pharmacological defatting of primary human hepatocytes which is non-toxic to cholangiocytes or intrahepatic endothelial cells

**DOI:** 10.1371/journal.pone.0201419

**Published:** 2018-07-25

**Authors:** Yuri L. Boteon, Lorraine Wallace, Amanda P. C. S. Boteon, Darius F. Mirza, Hynek Mergental, Ricky H. Bhogal, Simon Afford

**Affiliations:** 1 Liver Unit, Queen Elizabeth Hospital, University Hospitals Birmingham NHS Foundation Trust, Birmingham, United Kingdom; 2 National Institute for Health Research (NIHR) Birmingham Biomedical Research Centre, Institute of Immunology and Immunotherapy, College of Medical and Dental Sciences, University of Birmingham, Birmingham, United Kingdom; University College London, UNITED KINGDOM

## Abstract

**Introduction:**

Pharmacological defatting of rat hepatocytes and hepatoma cell lines suggests that the same method could be used to ameliorate macrovesicular steatosis in moderate to severely fatty livers. However there is no data assessing the effects of those drugs on primary human liver cells. We aimed to determine the effectiveness of a pharmacological cocktail in reducing the *in vitro* lipid content of primary human hepatocytes (PHH). In addition we sought to determine the cytotoxicity of the cocktail towards non-parenchymal liver cells.

**Methods:**

Steatosis was induced in PHH by supplementation with a combination of saturated and unsaturated free fatty acids. This was followed by addition of a defatting drug cocktail for up to 48 hours. The same experimental method was used with human intra-hepatic endothelial cells (HIEC) and human cholangiocytes. MTT assay was used to assess cell viability, triglyceride quantification and oil red O staining were used to determine intracellular lipids content whilst ketone bodies were measured in the supernatants following experimentation.

**Results:**

Incubation of fat loaded PHH with the drugs over 48 hours reduced the intracellular lipid area by 54%, from 12.85% to 5.99% (p = 0.002) (percentage of total oil red O area), and intracellular triglyceride by 35%, from 28.24 to 18.30 nmol/million of cells (p<0.001). Total supernatant ketone bodies increased 1.4-fold over 48 hours in the defatted PHH compared with vehicle controls (p = 0.002). Moreover incubation with the drugs for 48 hours increased the viability of PHH by 11%, cholangiocytes by 25% whilst having no cytotoxic effects on HIEC.

**Conclusion:**

These data demonstrate that pharmacological intervention can significantly decrease intracellular lipid content of PHH, increase fatty acids β-oxidation whilst being non-toxic to PHH, HIEC or cholangiocytes.

## Background

Hepatic steatosis results from the accumulation of triacylglycerol in the cytoplasm of hepatocytes which coalesce to form lipid droplets (LD). Large LDs that cause displacement of the cell nucleus are termed macrovesicular steatosis. Donor livers with macrovesicular steatosis are associated with significantly increased risk of early graft dysfunction after liver transplantation [1–4]. Intuitively defatting of steatotic donor livers could potentially improve both the organ utilisation and patient outcomes after transplantation. Using a static *in vitro* rat hepatocyte model where cells were loaded with fat, Nagrath *et al*. reported a reduction of 35% in the intracellular lipid content over 48 hours by supplementing media with a defatting cocktail consisting of peroxisome proliferator-activated receptor (PPAR)α ligands GW7647 and GW501516, pregnane X receptor (PXR) ligand Hypericin, the constitutive androstane receptor (CAR) ligand Scorparone, the glucagon mimetic cyclic adenosine monophosphate (cAMP) activator forskolin and the insulin-mimetic adipokine visfatin [5]. Thereafter the defatting cocktail was used in a model of ex-situ normothermic machine perfusion (NMP) of a whole steatotic rat liver and a reduction of 50% in the intracellular triglycerides levels was observed within 3 hours [5]. Subsequently the same protocol was applied to human hepatoma cells (HepG2 cells) by Yarmush *et al*. and similar findings were reported [6]. Consistently these studies demonstrate that the defatting cocktail increased mitochondrial beta-oxidation of fatty acids (FA) as represented by higher production of ketone bodies and upregulated the transcription of key enzymes involved with the exportation of intracellular lipids and oxidation of FA in the peroxisome [5, 6].

However, HepG2 cells do not accurately represent the response of primary human hepatocytes (PHH) to drugs or cellular stresses such as hypoxia and hypoxia/reoxygenation [7–9]. In particular HepG2 cells demonstrate a 90% reduction in cytochrome P450 expression [10]. Therefore before such defatting strategies can be considered for the use in machine perfusion of human donor livers, it is imperative that their efficacy and cytotoxicity be determined in models using human liver cells [11, 12]. In particular the cytotoxicity of the defatting drugs on other cell types within the liver such as intra-hepatic endothelial cells (HIEC) and cholangiocytes has not been assessed. The aim of the present study was to investigate the efficacy of this defatting drug cocktail on steatotic PHH and its cytotoxicity towards PHH, HIEC and cholangiocytes.

## Methods

### Study design

**Fig 1:**
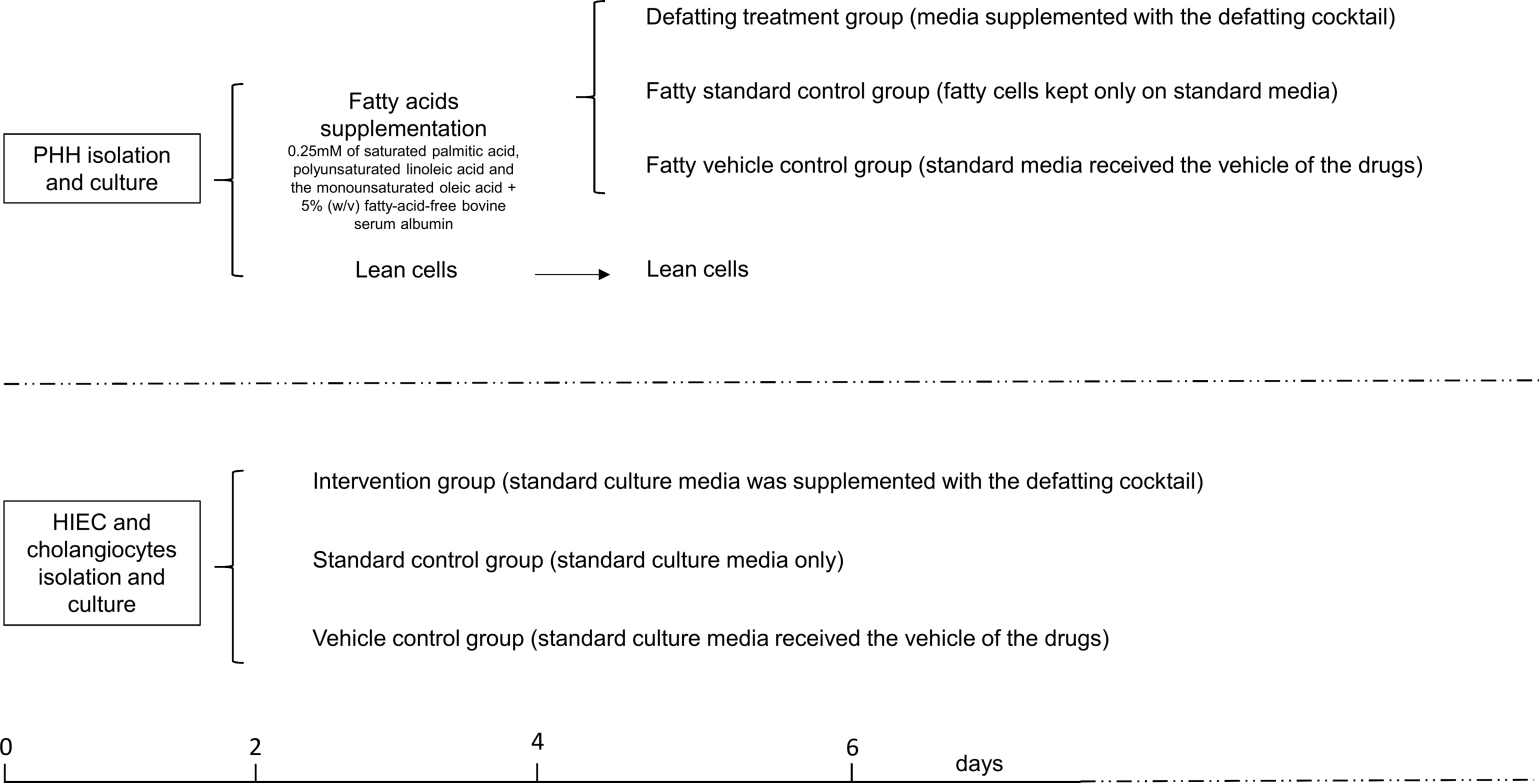
Study design. Series 1: Isolated primary human hepatocytes (PHH) were left in standard media for 2 days and then received media supplemented with fatty acids. After 2 days of fat loading the fatty PHH were allocated to the defatting treatment group where the media was supplemented with the defatting cocktail of drugs, and the control groups, the standard control group and the vehicle control group that received vehicle only. Lean hepatocytes were kept in standard culture conditions throughout the experimental period. The experimentation period lasted for two days thereafter. Series 2: Human intra-hepatic endothelial cells (HIEC) and cholangiocytes were immuno-magnetically separated with Dynabeads conjugated with cell-specific monoclonal antibody. The cells were left in culture for 2 days in standard media to reach confluence and then were allocated to the intervention group that received the defatting cocktail and the control groups, the standard control group and the vehicle control group that had the media supplemented with the vehicle only. The experimentation period lasts for two days thereafter.

Steatosis was induced in PHH by incubation of cells with FAs. Fatty loaded PHH were then incubated with a cocktail of defatting agents to test its cytotoxicity and effectiveness in reducing the intracellular lipid content. HIEC and cholangiocytes were also incubated with the defatting cocktail for 48 hours to assess the cytotoxicity of the cocktail. Three separate experiments were performed in quadruplicate. Fig 1 shows a schematic view of the study design.

### Source of liver tissues

The human cells were isolated from discarded donor livers. The organs were initially offered, accepted, and procured with the intention of clinical transplantation. They were then declined by all UK transplant centres and offered for research by the National Health Service Blood and Transplant (NHSBT) coordinating office. Specialist nurses in organ donation obtained consent to use donor tissue for research as part of the consent process for standard clinical organ donation. None of the donor organs were from a vulnerable population and all next of kin provided written informed consent that was freely given. Ethical approval for the study was granted by the London-Surrey Borders National Research Ethics Service committee as well as Loco-Regional and NHSBT Ethics Committees (reference 13/LO/1928 and 06/Q702/61). Cells were isolated from three donors after brain stem death declined for transplantation because of logistics and were preserved by static cold storage in University of Wisconsin preservation fluid.

### PHH cell isolation

A published collagenase perfusion technique was employed for PHH isolation from liver wedges [13]. Briefly liver was digested and centrifuged to isolate PHH. These cells were resuspended and then plated on 24-well plates previously coated with rat tail collagen type 1 at a density of 3x10^5^ cells/well in Dulbecco’s Modified Eagle’s Medium (DMEM) (Catalogue number [CN]:41965–039; Gibco laboratories, Gaithersburg, MD, USA) supplemented with 10% fetal calf serum (FCS) (CN:10270106; Gibco) and 5% glutamine/penicillin/ streptomycin (GPS) (CN:10378016; Gibco). After 2 hours the cells were washed with Phosphate-buffered saline (PBS) (CN:10010023; Gibco) and the media changed to our standard medium for PHH culture, constituted of Arginine-/Glutamine-free Williams E (CN:12551032; Gibco) with 1% GPS, hydrocortisone (2μg/ml) (H4001; Sigma-Aldrich, St. Louis, MO., USA), insulin (0.124 U/ml) (I2643; Sigma-Aldrich) and L-ornithine (400μM) (O6503; Sigma-Aldrich), subsequently the cells were kept at 37°C in 95% air/ 5% CO_2_.

### *In vitro* steatosis induction for PHH

The standard media for PHH culture was supplemented with a combination of FAs in order to promote increases in the intracellular triglyceride levels stocked as LDs, as previously described [14]. This fatting media consisted of the saturated palmitic acid (P0500; Sigma-Aldrich), polyunsaturated omega-6 linoleic acid (L5900; Sigma-Aldrich) and the monounsaturated omega-9 oleic acid (O1257; Sigma-Aldrich) all at a final concentration of 0.25mM. This concentration was determined by performing cytotoxicity titration experiments prior to institution of the full experimental protocol. A supplement of 5% fatty-acid-free bovine serum albumin weight/volume (BSA) (A3803; Sigma-Aldrich) was added as a protein carrier. The media was changed daily and the steatosis induction period was 48 hours. The lean control group was incubated with standard media only throughout the experimental period.

### Defatting medium for PHH

Following steatosis induction the fatting media was removed and cells washed with PBS. Experiments were then performed on 4 distinct groups: (1) the fatty vehicle control group, which received the cell type specific standard media described above plus the vehicle dimethylsulfoxide (DMSO) <0.1% v/v (D2438; Sigma-Aldrich) used for drugs dilution, without any drug or fatty acid supplement; (2) the fatty standard control group, which received only the standard culture media; (3) the defatting treatment group, which had the media supplemented with the combination of defatting drugs (0.01 mM glucagon mimetic and cAMP activator forskolin [F6886; Sigma-Aldrich], 0.001 mM PPAR α ligand GW7647 [G6793; Sigma-Aldrich], 0.01 mM PXR ligand hypericin [56690; Sigma-Aldrich], 0.01 mM CAR ligand scoparone [254886; Sigma-Aldrich], 0.001 mM PPAR δ ligand GW501516 [SML1491; Sigma-Aldrich], 0.4 ng/ml adipokine visfatin [SRP4908; Sigma-Aldrich] and 0.8 mM L-carnitine [C0283; Sigma-Aldrich]); and, (4) lean cells that were kept on standard media throughout. The defatting mixture of drugs was tested previously in rat hepatocytes and HepG2 cells [5, 6, 15]. All groups had the media changed and sampled after 24 hours and 48 hours of treatment and the cells harvested for intracellular lipids quantification.

### Isolation and culture of primary cholangiocytes and HIEC

HIEC and cholangiocytes were isolated from human liver tissue using Collagenase Type 1A (C9891; Sigma-Aldrich) digestion for 1 hour at 37°C. The resulting cell suspension was then sieved through a fine mesh, separated on a 33%/77% Percoll density gradient and cells retrieved from the interphase. This interphase mixed population of cells were then diluted in PBS, centrifuged and further immuno-magnetically separated with Dynabeads conjugated with cell-specific monoclonal antibody (anti-cluster of differentiation 31 [CD31] to purify HIEC [M0823, monoclonal mouse antibody anti-CD31, clone JC70A; Dako, Denmark] or anti-epithelial cell adhesion molecule [130-080-301, monoclonal mouse antibody, CD326, EpCAM-FITC; Miltenyi Biotec, Bergisch, Germany] to purify cholangiocytes). The extracted cholangiocytes and HIEC were then plated on 96-well plates previously coated with rat tail collagen type 1; Cholangiocytes in DMEM 10% FCS supplemented with 5% GPS and the HIEC in Human Endothelial-Serum Free Media (CN:11111044; Gibco) supplemented with 10% heat-inactivated human serum (CR100; TCS Biologicals, Buckingham, UK) with 5% GPS. After an interval of 12 hours the media was changed to our standard specific culture medium. For cholangiocytes it was constituted of DMEM/ HAMS F-12 nutrient mixture (CN:21331–020; Gibco) 1:1 v/v, 5% GPS, hydrocortisone (0.4 μg/ml), cholera toxin (10 ng/ml) (C8052; Sigma-Aldrich), triiodothyronine (T3) (2x10^-9^ mol/L) (T6397; Sigma-Aldrich), insulin (5 μg/ml), hepatocyte growth factor (10 ng/ml) (CN:100–39; Peprotech, Rocky Hill, NJ, USA) and epidermal growth factor (10 ng/ml) (CN:100–61; Peprotech). For HIEC Human Endothelial-Serum Free Media supplemented with 10% heat-inactivated human serum, vascular endothelial growth factor (10 ng/ml) (CN:100-20C; Peprotech) and hepatocyte growth factor (10 ng/ml) was used. After isolation the cells were kept in an incubator at 37°C in an atmosphere of 95% air/ 5% CO_2_.

Cholangiocytes and HIEC were cultured for 48 hours to reach confluence. After this period the culture media was changed and the experimental groups assigned to the various cells. The intervention group received the standard culture medium supplemented with the defatting drugs (0.01 mM forskolin, 0.001 mM GW7647, 0.01 mM hypericin, 0.01 mM scoparone, 0.001 mM GW501516, 0.4 ng/ml visfatin and 0.8 mM L-carnitine). The control group was split into two, one that received vehicle (DMSO <0.1% v/v) and a second one that received only the standard culture medium.

### Cell viability assessment

Cell viability was assessed using MTT (3-(4,5-dimethylthiazol-2-yl)-2,5-diphenyltetrazolium bromide) assay (M5655; Sigma-Aldrich). MTT is initially a yellow-coloured solution which turns purple formazan after reduction by reductase enzymes found within mitochondria of viable cells. DMSO is added to dissolve the purple formazan into a coloured solution. The readout was the difference between the values of the absorbance readings at 570 and 690 nm on a plate reader. The amount of purple formazan produced by control cells allows comparisons between the effects of different treatments. MTT assay was used to assess the cytotoxicity of the mixture of drugs and of the media supplemented with FAs.

### Oil red O staining

Oil Red O staining was employed for quantifying LDs in the cytoplasm of the cells (O0625; Sigma-Aldrich). Following experimentation cells were fixed with buffered formaldehyde and the staining was carried out as previously described [16]. Mayer’s haematoxylin was used for the nuclear counter stain.

For quantification of staining in PHH four high power field (HPF) images from each experiment were selected. Positive areas of staining were calculated by a system of colour differentiation and the result expressed in percentage of the total area of the image using ImageJ, U. S. National Institutes of Health, Bethesda, Maryland, USA.

### Intracellular triglyceride quantification

At the end of the incubation period, cells were washed with PBS and harvested with gentle scraping. Intracellular lipids were retrieved using the detergent TERGITOL™ Type NP-40 (NP40S; Sigma-Aldrich) followed by lipase incubation to break down triglycerides into FAs and glycerol. Intracellular triglyceride was measured using a colorimetric assay (ab65336; Abcam, Cambridge, MA, USA) based on a reaction of glycerol oxidation producing colour. The concentration of triglycerides was normalised to control and expressed as nmol per million of cells.

### Ketone bodies measurement

Ketone bodies were quantified in cell supernatants using a commercially available kit (MAK134; Sigma-Aldrich) as per the manufacturer’s instructions and detect 3-hydroxybutyric acid (BOH) and acetoacetic acid (AcAc).

### Statistical analysis

Continuous variables were expressed as median/ interquartile range or range and categorical variables as absolute number/ frequency (%). Comparison between groups was performed using Mann–Whitney U test or two tailed t-test for continuous variables. The statistical level of significance was p<0.05. GraphPad Prism version 6.04 for Windows, GraphPad Software, La Jolla California USA was used for statistical analysis and graph creation.

## Results

### Induction of steatosis in PHH

**Fig 2:**
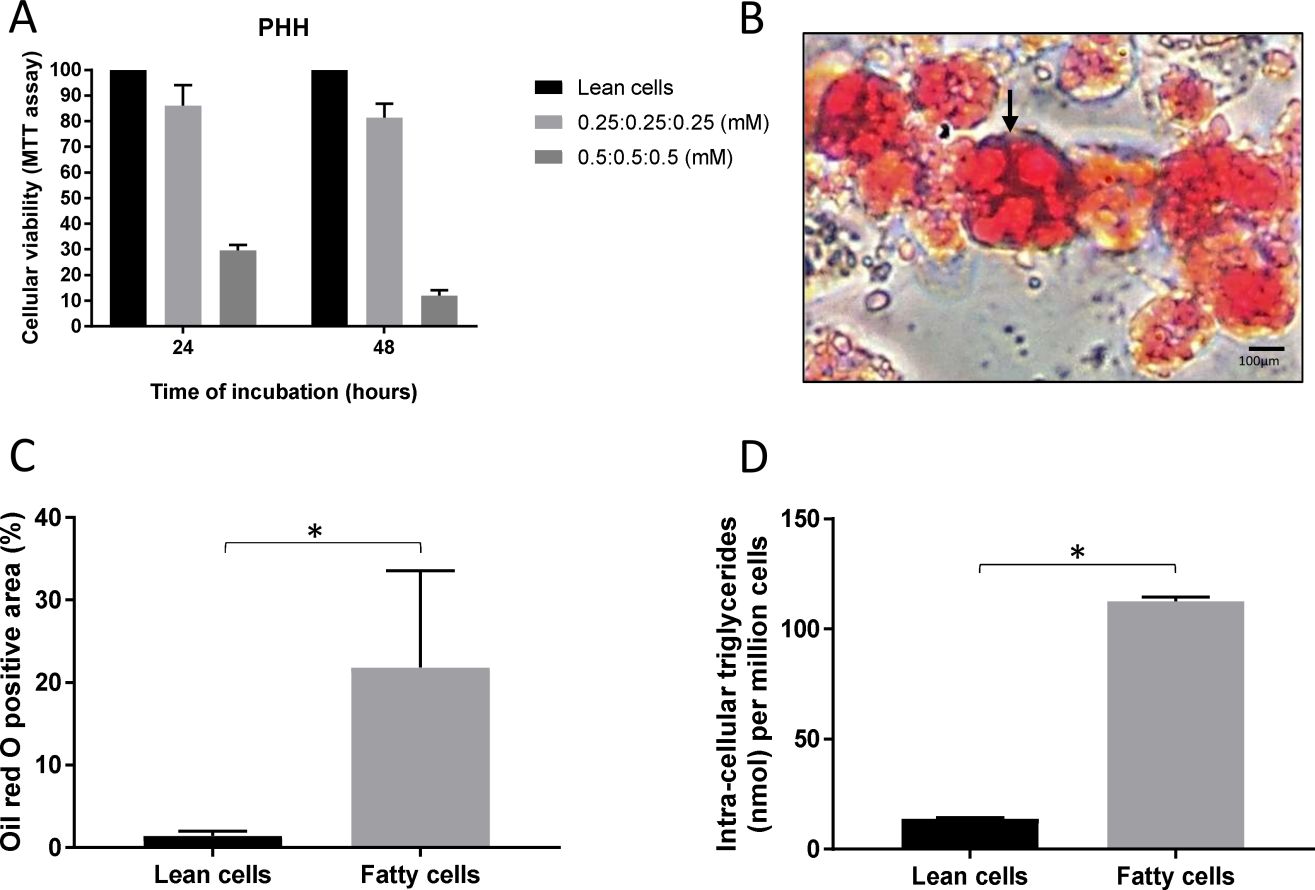
Results of fat loading of primary human hepatocytes. Panel A: The supplementation of the media with the combination of fatty acids resulted in a cell viability rate of 81% after 48 hours of incubation. Panel B: Oil red O staining image of PHH at the end of the fat loading period. There is predominance of large lipid droplets displacing the nucleus of the cells to the periphery (black arrow). Panel C: At the end of 48 hours of fatting load there was a significant increase of 14-fold of the positive area of oil red O. Panel D: Intracellular triglycerides increased 8-fold within 48 hours of incubation with fatty acids. Data report the median of three separate experiments performed in quadruplicate and errors bars the interquartile range. Comparisons performed using two-tailed t-test. * = p<0.05.

Incubation of PHH for 48 hours with media supplemented with the combination of FAs increased the median intracellular triglyceride concentration approximately 8-fold, from 14.01 (range 13.69–14.02) to 112.64 (111.65–113.30) nmol/million of cells (p<0.001). The PHH oil red O staining area increased significantly from 1.4% (0.9–1.7) to 21.8% (14.7–32.0) (approximately 14-fold) (p<0.05). The cellular viability of PHH after 48 hours of incubation with the media supplemented with FAs using the MTT-assay was 81% (range 76–87%) in comparison with lean cells kept in the standard media throughout. The results from the steatosis induction period are in Fig 2.

### Defatting of PHH

#### Oil red O analysis

**Fig 3:**
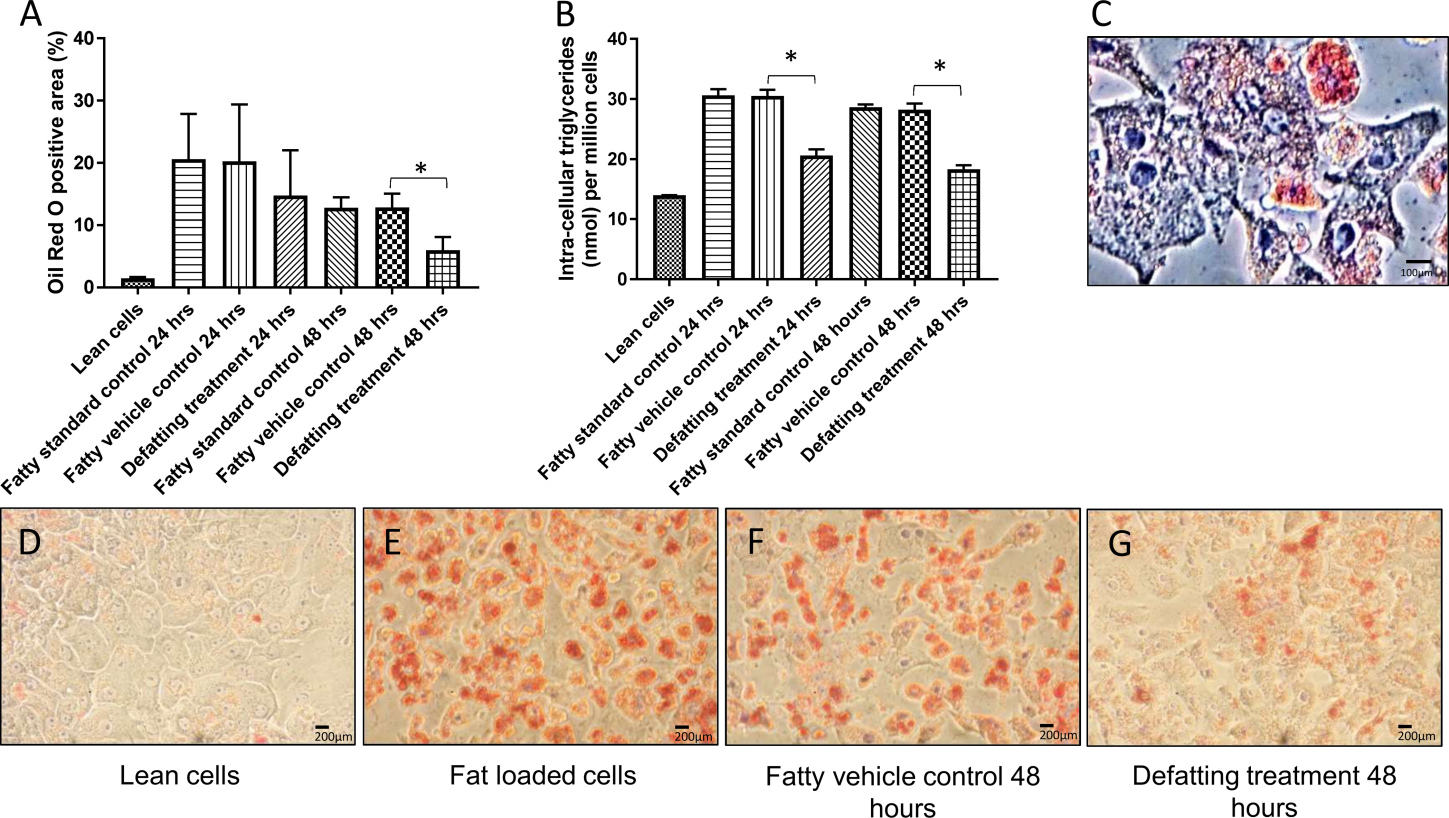
Defatting of fat loaded primary human hepatocytes (PHH). Panel A: The positive are of oil red O of the defatting treatment group was reduced by 28% in comparison with the vehicle control group over 24 hours and 54% over 48 hours. Panel B: Intracellular triglyceride levels of the defatting treatment group were reduced by 32% within 24 hours of treatment and 35% within 48 hours, in comparison with the fatty vehicle control group. Panel C: Oil red O staining picture of PHH of the defatting group at the end of the 48 hours of treatment. There is a predominance of small lipid droplets in the cytoplasm of the cells and the nucleus is in its usual position. Series 2: shows a series of oil red O staining pictures from PHH in culture at different time points of the experiments. Panel D shows lean cells in culture, after the incubation with fatty acids they become loaded with fat (panel E). Those fat loaded PHH were then incubated with only the vehicle of the drugs for 48 hours and the lipid content decreased over time (panel F) or had the defatting treatment that showed the significant higher decrease in the area of oil red O (panel G). Data report the median of three separate experiments performed in quadruplicate and errors bars the interquartile range. Comparisons performed using two-tailed t-test. * = p<0.05.

Digital analysis of oil red O staining of cytoplasmic LDs demonstrated that the combination of drugs lead to a decrease from 28% [20.27%(14.67–32.03%) to 14.77%(11.70–22.05%), p = 0.315] in median positive area of oil red O staining within 24 hours of treatment and 54% within 48 hours [12.85% (11.07–15.80%) to 5.99% (4.24–8.61%), p = 0.002] compared to the fatty vehicle control hepatocytes alone (Fig 3). The positive area of oil red O was comparable between the fatty vehicle control cells and the fatty standard control cells after 24 hours [20.27%(14.67–32.03) vs. 19.98%(15.03–31.70), p>0.999] and 48 hours of treatment (12.85% (11.07–15.80%) vs. 13.04% (12.01–16.02), p>0.999]. The same pattern was seen for the other parameters analysed therefore comparisons with the defatting group were made using the fatty vehicle control group. In addition to the decrease in the total area of cytoplasmic LDs (expressed as percentage oil red O positive area) in the defatting group, it morphologically appeared to switch from a macrovesicular to a microvesicular appearance (Fig 3).

#### Intracellular triglyceride quantification

Treatment with the defatting cocktail decreased the median concentration of intracellular triglycerides by 32%, from 30.51 nmol/million of cells (range 30.18–31.50) in the fatty vehicle control group to 20.61 nmol/million of cells (range 20.28–21.60) in the defatting group within 24 hours, p = 0.012. After 48 hours it reduced 35%, from 28.24 nmol/million of cells (range 26.88–29.24) to 18.30 nmol/million of cells (range 18.30–18.96) (p<0.001). Intracellular triglyceride concentration reduced over time once the cells were removed from the fatting media by approximately 5-fold within 24 hours (Fig 3). There was no difference between intracellular triglycerides levels of the fatty control vehicle alone and the fatty standard control group (p>0.999).

#### Fatty acids β-oxidation induction

**Fig 4:**
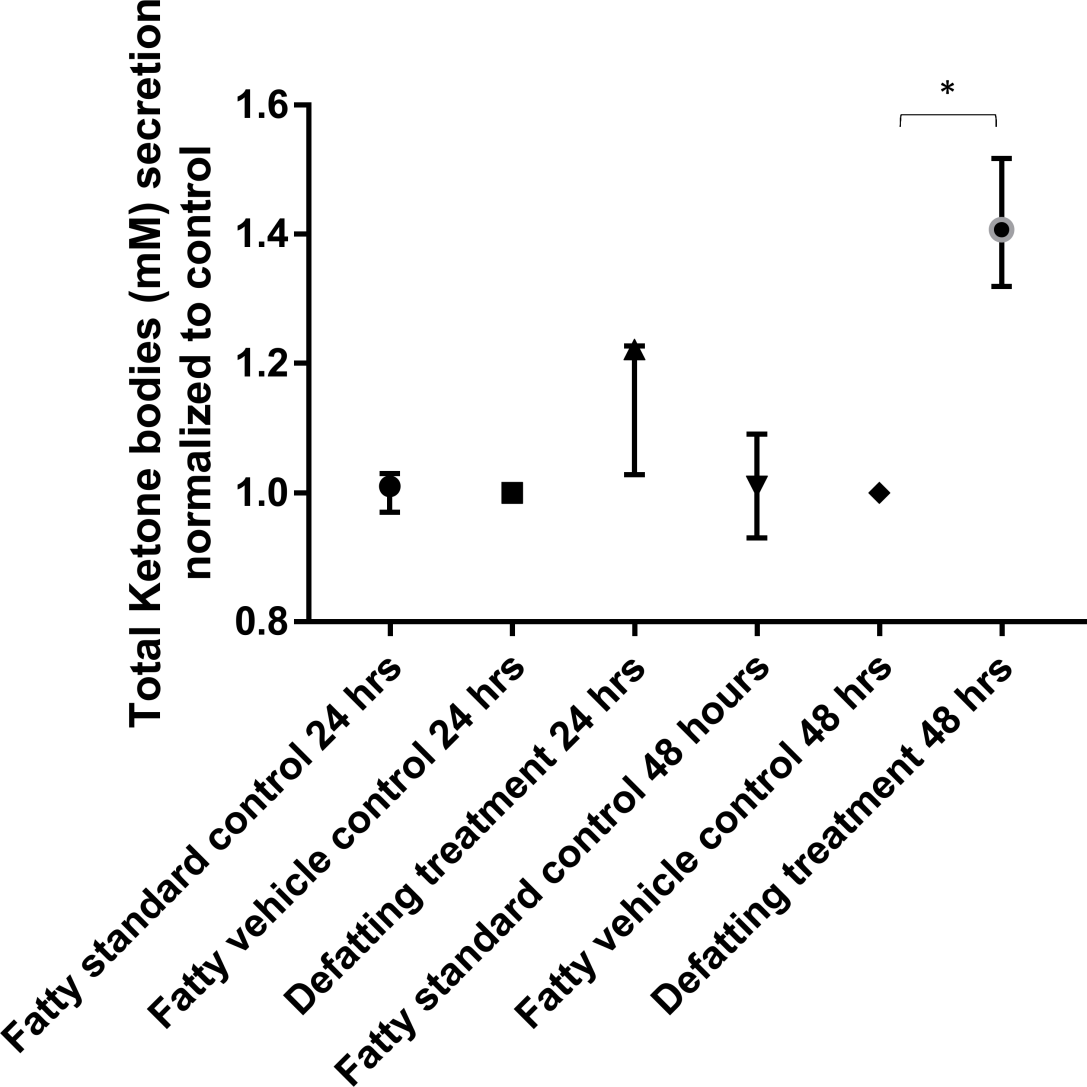
Release of total ketones in the supernatants. Fat loaded primary human hepatocytes that had the defatting treatment showed an increase in cell culture supernatant levels of total ketone bodies of 1.22-fold over 24 hours and 1.40-fold over 48 hours. Data reports the median of three separate experiments performed in quadruplicate and errors bars the interquartile range. Comparisons performed using two-tailed t-test. * = p<0.05.

The defatting cocktail induced a median increase in the release of total ketone bodies in the supernatant of PHH of 1.22-fold (range 1.02–1.26) (p = 0.070) after 24 hours of treatment and 1.40-fold within 48 hours (range 1.31–1.52) (p = 0.002) (Fig 4) when compared to the fatty vehicle control group alone at each respective time point. The release of total ketone bodies in the supernatant was similar between both control groups.

### Impact of the defatting cocktail on cell viability

#### Effects on PHH

**Fig 5:**
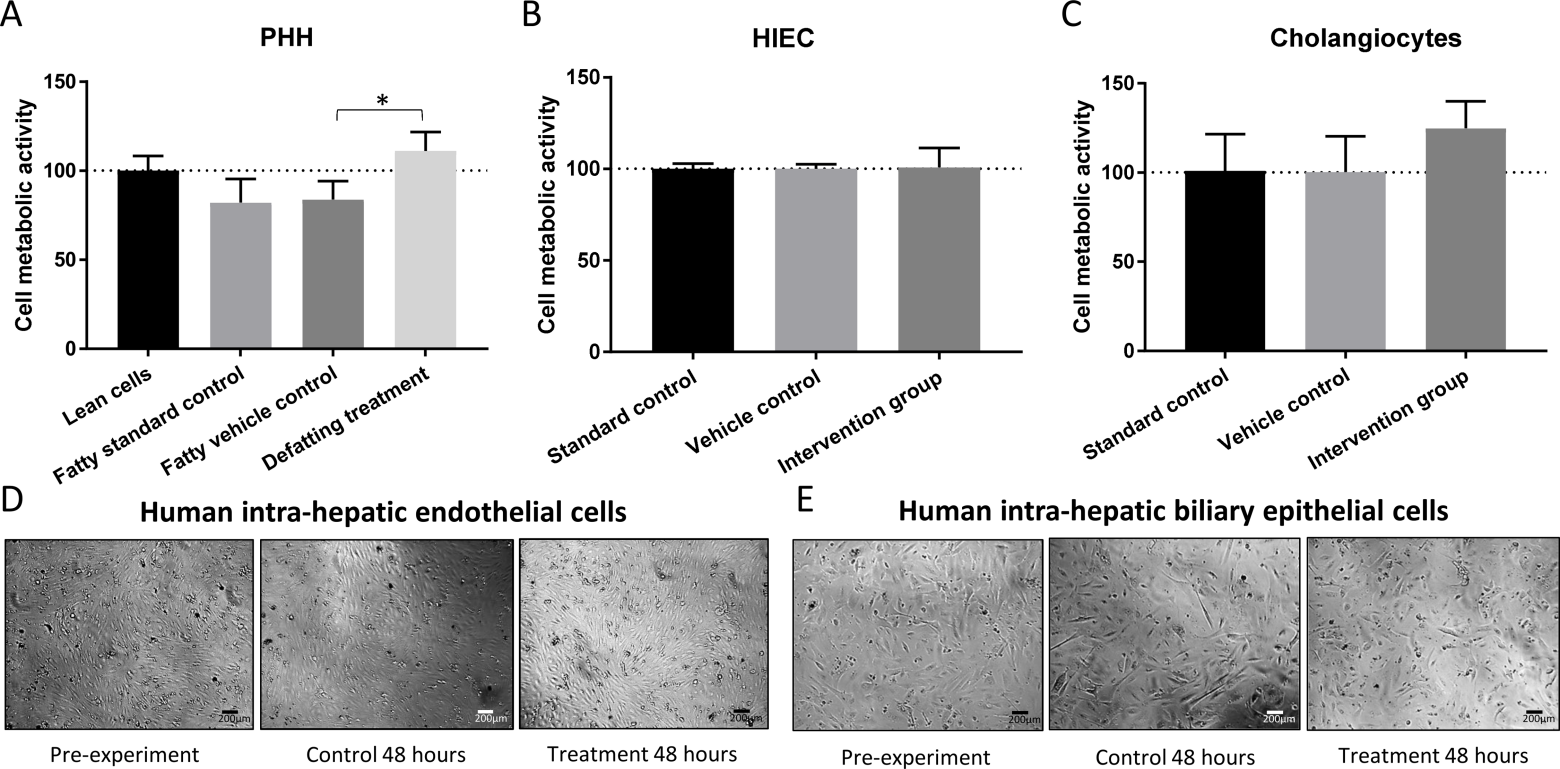
Assessment of the cytotoxicity of the defatting cocktail to human cells of the liver via MTT assay. Panel A: the toxicity of the defatting cocktail was tested in primary human hepatocytes and results showed a significant improvement of 11% in viability of the defatting treatment group compared with the fatty vehicle control group. Panel B: Treatment of human intra-hepatic endothelial cells (HIEC) with the drugs had no effect on cell viability compared with the control groups. Panel C: treatment of cholangiocytes with the defatting cocktail did not demonstrate any cytotoxic effect to the cells and indicated a slight improvement in viability compared to the control groups. Panels D and E: Phase contrast microscopy showing representative images of HIEC (Panel D) and cholangiocytes (Panel E) at different time points. No gross modifications in cell integrity were observed in either cell type which was consistent and supportive of the MTT data. Data report the median of three separate experiments performed in quadruplicate and errors bars the interquartile range. Comparisons performed using two-tailed t-test. * = p<0.05.

After 48 hours of treatment with the defatting cocktail MTT assay showed an increase in the viability of PHH of 11% (6–15%) in comparison with the fatty vehicle control cells (p = 0.048). Cellular viability was similar between the control groups. Phase contrast light microscopy suggested that the defatted hepatocytes were more adherent and spread on the wells in comparison with the fatty vehicle control cells which continued to die (Fig 5).

#### Effects of the defatting cocktail on other liver cell types

Incubation of cholangiocytes and HIEC with the defatting cocktail was not cytotoxic. There was no difference in viability between HIEC in the treated group and the vehicle control group after 48 hours of incubation (100% [97–101] vs. 100% [90–113], p>0.999). For cholangiocytes the supplementation of the media with those drugs improved cellular viability in 25% although the difference did not reach statistical significance (125% [75–166%] vs. 100% [57–116], p = 0.413). Detailed data is presented in Fig 5.

## Discussion

Defatting of steatotic rat livers using a cocktail of drugs in a model of ex-situ normothermic machine perfusion was shown to be feasible and a potentially promising translational approach to improve the utilisation of steatotic donor livers for transplantation [2]. However before considering the translational application of such interventions to whole human donor livers, the efficacy and cytotoxicity of these agents to human liver cells needed to be evaluated since there is inherent variability in responses to drugs between species and different cell lines [7–9]. Our study demonstrates for the first time using PHH that the defatting cocktail was able to reduce its lipid content *in vitro* enhancing beta-oxidation of FAs. Moreover we have shown that the drugs were not toxic to PHH, HIEC or cholangiocytes. The latter being crucial in the clinical setting where these cells are targets in ischaemia-reperfusion injury and ischaemic cholangiopathy [17, 18].

Steatosis is a frequent reason for livers being deemed non transplantable [19]. This is because these organs are more susceptible to ischaemic injury during cold preservation and thereafter are at a high risk of graft dysfunction after transplantation [1–4, 20, 21]. Current strategies for transplantation of steatotic deceased donor livers rely mainly on the prevention of additional risk factors, such as limiting cold ischaemic times, using low risk donors and selecting low risk recipients [22]. An intervention which may decrease post-transplant complications for such livers has been described in the context of living donation. Living donors that had their diet supplemented with Ω-3 FAs one month before organ procurement appears to be associated with fewer post-operative complications. Although this approach shows promise, it is not applicable in the context of deceased donor organs [22, 23].

Nagrath *et al*. 2009 tested a cocktail of drugs in an *in-vitro* model of steatotic rat hepatocytes and showed a decrease in the intracellular triglyceride levels of 31% within 48 hours [5]. This cocktail was tested then by Yarmush *et al*. 2016 in an *in vitro* model of hepatoblastoma cells (HepG2) loaded with lipids via FAs supplementation. The combination of drugs promoted a reduction of 83% in intracellular triglycerides within 48 hours of treatment under hyperoxic conditions [6]. We have employed the same cocktail of drugs in our experiments and in PHH we found a reduction of 35% over the same time period. This reduction was more significant considering the positive area occupied by LDs (54%). Our data suggests that this finding is likely to be related to the reported decrease in size of the LDs gaining appearance of microvesicular steatosis. This decrease in macrovesicular steatosis was associated with a continuous increase in the production of ketone bodies with the defatting cocktail in comparison with fatty control cells further than 24 hours, a product of incomplete oxidation of FAs in the mitochondria.

The defatting cocktail consists of: nuclear ligands for peroxisome proliferator-activated receptors (PPAR α- ligand GW7647; and, PPAR δ- ligand GW501516) to stimulate the transcription of lipid oxidation/exportation factors [24]; an insulin-mimetic adipokine visfatin associated with lowering triglyceride levels in the liver [25]; forskolin a glucagon mimetic molecule known to stimulate cyclic AMP-driven β-oxidation of lipids and ketogenesis [26]; pregnane X Receptor (PXR) ligand hypericin that is reported to improve the transcription of the cytochrome P450 (CYP) 3A4 monooxygenase which can increase metabolism of a range of drugs in hepatocytes [27]; constitutive androstane receptor (CAR) ligand scoparone (6,7-dimethoxycoumarin) that acts to promote transcription of beta-oxidation enzymes, such as carnitine palmitoyltransferase 1 [28], and finally a supplement of L-carnitine, fundamental in the transport of FAs across the inner mitochondrial membrane [29].

In brief, forskolin activating glucagon membrane receptors can stimulate the adenosine monophosphate (cAMP)–protein kinase A pathway that regulates the trafficking of cytoplasmic lipases to the surface of LDs [30, 31]. The glycerol and FAs released from the breakdown of LDs could potentially not only serve as substrates for the cell metabolism but also as ligands to nuclear receptor (peroxisome proliferator receptor [PPAR] and liver X receptors [LXR]) increasing the transcription of enzymes involved in the catabolism of FAs in the mitochondria and peroxisome [32, 33]. The other drugs (GW7647, GW501516, hypericin, scorparone) also act as ligands to other nuclear receptors (pregnane X receptors and androstane receptors) boosting the transcription of key enzymes in lipid metabolism [34, 35]. Cytosolic fatty acid reacts with ATP generating fatty acyl-CoA. Acyl-CoA in turn reacts with apolipoprotein B to generate lipoproteins to be exported from the cell and/or reacts with the hydroxyl group of carnitine via carnitine palmitoyltransferase 1. Acyl-carnitine is transported inside the mitochondria by a Carnitine-acyl-CoA transferase and a carnitine is transferred outside. Acyl-CoA is processed by β-oxidation then allowing ketogenesis or complete oxidation via the Krebs cycle and the electron transport chain with the production of adenosine triphosphate (ATP) [36]. Therefore acting through different pathways this combination of drugs accelerates the process of intracellular triglycerides exportation and improves mitochondrial oxidation of FAs [5, 37]. Hence the defatting cocktail not only reduces intracellular LD content but it also serves to enhance lipid metabolism and potentially increase intracellular ATP. This increased ATP content can potentially improve the poor outcome of steatotic livers during either machine perfusion or after clinical transplantation as loss of energy reserves is an important reason for graft dysfunction [38].

As already stated, experiments testing the defatting cocktail with rat hepatocytes and HepG2 cells do not wholly reflect the responses of PHH. For example, the expression of enzymes involved in drug metabolism as cytochrome P450 is variable between these cells and PHH [7, 10]. Consequently such experiments although informative, may underestimate the real cytotoxicity or metabolic effect of therapeutic interventions in human livers [7, 8, 10, 39]. Therefore PHH remain the choice of cells for the study of cytotoxicity and the resultant metabolic effect of drugs in human livers [11, 12, 39].

The present study to the best of our knowledge is the first to examine the cytotoxicity and metabolic effect of a combination of drugs intending to promote defatting of fat loaded PHH in an *in vitro* model. It was shown that the defatting cocktail effectively decreased the lipid content of PHH *in vitro*, improving cellular viability and mitochondrial oxidation of FAs. No less importantly, we have tested the cytotoxicity of the drugs to HIEC and cholangiocytes. The information that the drugs are not toxic to these cells is an important and reassuring step before moving towards translational experiments for the delivery of the defatting cocktail to whole human donor livers during extra-corporeal normothermic machine perfusion.

Despite the experimental evidence discussed, defatting of human livers remains challenging and under researched. One limitation of our study is that we have not explored what could be the impact of flow and the effect of ischaemia-reperfusion on the defatting process of PHH. It was suggested recently in the literature by Yarmush *et al*. 2017 that cultured HepG2 cells can have the time for defatting shortened from 48 hours to 4–6 hours when submitted to conditions of flow using this cocktail of drugs [37]. In addition machine perfusion and/or clinical transplantation of livers will involve a period of ischaemia and this may have an effect upon defatting [38].

## Conclusion

Using an *in vitro* model of PHH, our study demonstrates for the first time that pharmacological interventions can be used to lower intracellular triglycerides stores and promote higher rate of FAs mitochondrial β-oxidation. Additionally the drugs were shown to be not toxic to PHH, HIEC and cholangiocytes. Hence, the present study supports future translational experiments involving the described defatting cocktail in steatotic human livers.
